# The built environment and active transportation safety in children and youth: a study protocol

**DOI:** 10.1186/s12889-019-7024-6

**Published:** 2019-06-11

**Authors:** Brent E. Hagel, Alison Macpherson, Andrew Howard, Pamela Fuselli, Marie-Soleil Cloutier, Meghan Winters, Sarah A. Richmond, Linda Rothman, Kathy Belton, Ron Buliung, Carolyn A. Emery, Guy Faulkner, Jacqueline Kennedy, Tracey Ma, Colin Macarthur, Gavin R. McCormack, Greg Morrow, Alberto Nettel-Aguirre, Liz Owens, Ian Pike, Kelly Russell, Juan Torres, Donald Voaklander, Tania Embree, Tate Hubka

**Affiliations:** 1 Departments of Pediatrics and Community Health Sciences, Cumming School of Medicine, University of Calgary, C4-434, Alberta Children’s Hospital, 28 Oki Drive NW, Calgary, Alberta T3B 6A8 Canada; 2 Faculty of Health, 337 Norman Bethune College, York University, BC Keele Campus, Toronto, Ontario M3J 1P3 Canada; 3 Hospital for Sick Children, 555 University Avenue, Room S – 107, Toronto, Ontario M5G 1X8 Canada; 4 Parachute Canada, 150 Eglinton Ave East, Suite 300, Toronto, Ontario M4P 1E8 Canada; 5 Institut National de la Recherche Scientifique, 385, rue Sherbrooke Est, Montréal, H2X 1E3 Québec Canada; 6 Faculty of Health Sciences, Simon Fraser University, 8888 University Drive, Burnaby, V5W 1G1 British Columbia Canada; 7 Health Promotion, Chronic Disease and Injury Prevention, Public Health Ontario, 480 University Ave, Toronto, Ontario M5G 1V2 Canada; 8 Child Health Evaluative Sciences, Hospital for Sick Children; Dalla Lana School of Public Health, Epidemiology Division, University of Toronto, 555 University Ave, Toronto, Ontario M5G 1X8 Canada; 9 Injury Prevention Centre, University of Alberta, 4075 RTF, 8308 114 St NW, Edmonton, Alberta T6G 2E1 Canada; 10 Department of Geography Planning, University of Toronto, 3359 Mississauga Road N, Mississauga, Ontario L5L 1C6 Canada; 11 Sport Injury Prevention Research Centre, University of Calgary, 2500 University Dr NW, Calgary, Alberta T2N 1N4 Canada; 12 School of Kinesiology, University of British Columbia, Lower Mall Research Station, 2259 Lower Mall, Rm 337, Vancouver, British Columbia V6T 1Z4 Canada; 13 Green Communities Canada, 416 Chambers Street, 2nd Floor, Peterborough, Ontario K9H 3V1 Canada; 14 The George Institute for Global Health; School of Public Health and Community Medicine, University of New South Wales, Level 5, 1 King St, Newtown, New South Wales 2042 Australia; 15 Hospital for Sick Children Research Institute, 686 Bay Street, Toronto, Ontario M5T 3M6 Canada; 16 Cumming School of Medicine, University of Calgary, 3300 Hospital Dr. NW, Calgary, Alberta T2N 4N1 Canada; 17 College of Environmental Design, University of California, 230 Wurster Hall, Berkeley, California 94720 USA; 18 Departments of Pediatrics and Community Health Sciences, Cumming School of Medicine, University of Calgary, C4-435, Alberta Children’s Hospital, 28 Oki Drive NW, Calgary, T3B 6A8 Alberta Canada; 19 Office of Traffic Safety, Alberta Transportation, Room 109, Main Floor Twin Atria Building, 4999 - 98 Avenue NW, Edmonton, Alberta T6B 2X3 Canada; 20 Department of Pediatrics, University of British Columbia; BC Injury Research and Prevention Unit, BC Children’s Hospital Research Institute, F508, 4480 Oak St, Vancouver, V6H 3V4 British Columbia Canada; 21 Department of Pediatrics and Child Health, University of Manitoba, 656-715 McDermont Avenue, Winnipeg, Manitoba R3E 3P4 Canada; 22 Faculté de l’aménagement, Université de Montréal, C.P. 6128, succ. Centre-ville, Montréal, Québec H3C 3J7 Canada; 23 Acadia University, Box 48, 32 Acadia Avenue, Wolfville, Nova Scotia B4P 2R6 Canada; 24 Departments of Pediatrics and Community Health Sciences, Cumming School of Medicine, University of Calgary, C4-433-03, Alberta Children’s Hospital, 28 Oki Drive NW, Calgary, Alberta T3B 6A8 Canada

**Keywords:** Built environment, Active transportation, Injury prevention, Children

## Abstract

**Background:**

Active transportation, such as walking and biking, is a healthy way for children to explore their environment and develop independence. However, children can be injured while walking and biking. Many cities make changes to the built environment (e.g., traffic calming features, separated bike lanes) to keep people safe. There is some research on how effective these changes are in preventing adult pedestrians and bicyclists from getting hurt, but very little research has been done to show how safe various environments are for children and youth. Our research program will study how features of the built environment affect whether children travel (e.g., to school) using active modes, and whether certain features increase or decrease their likelihood of injury.

**Methods:**

First, we will use a cross-sectional study design to estimate associations between objectively measured built environment and objectively measured active transportation to school among child elementary students. We will examine the associations between objectively measured built environment and child and youth pedestrian-motor vehicle collisions (MVCs) and bicyclist-MVCs. We will also use these data to determine the space-time distribution of pedestrian-MVCs and bicyclist-MVCs. Second, we will use a case-crossover design to compare the built environment characteristics of the site where child and youth bicyclists sustain emergency department reported injuries and two randomly selected sites (control sites) along the bicyclist’s route before the injury occurred. Third, to identify implementation strategies for built environment change at the municipal level to encourage active transportation we will conduct: 1) an environmental scan, 2) key informant interviews, 3) focus groups, and 4) a national survey to identify facilitators and barriers for implementing built environment change in municipalities. Finally, we will develop a built environment implementation toolkit to promote active transportation and prevent child pedestrian and bicyclist injuries.

**Discussion:**

This program of research will identify the built environment associated with active transportation safety and form an evidence base from which municipalities can draw information to support change. Our team’s national scope will be invaluable in providing information regarding the variability in built environment characteristics and is vital to producing evidence-based recommendations that will increase safe active transportation.

## Background

The Convention on the Rights of the Child states that “…children have the right to the highest attainable level of health and the right to a safe environment, free from injury and violence” [1]. Injuries are the most common cause of death and disability in children and youth and the majority of serious injuries are preventable [1]. Motor vehicle collisions (MVCs), including collisions with pedestrians and bicyclists, are the leading cause of paediatric injury and death. Andrew Howard, a leading Canadian surgeon, and injury prevention expert has argued that: “By giving priority to automotive over pedestrian transportation, we have allowed road traffic to become the leading cause of death among our children” [2]. Until we start to address motor vehicle related pedestrian and bicycling injury via primary prevention strategies, such as modifying urban form (i.e., the built environment) to improve the support and safety of active transportation, these disturbing facts will not change. This research program focuses on the built environment and active transportation safety in children and youth. A built environment that promotes active transportation is necessary to meet both health goals (health promotion, injury prevention) and health equity goals (valuing children, reducing socioeconomic health and injury gradients).

Active transportation by children and youth increases their physical activity, which improves physical and mental health, maintenance of a healthy body weight, academic performance, motor skill development & physical literacy [3] [4]. Furthermore, physical activity habits and attitudes formed in childhood and adolescence are usually carried into adulthood [3–6]. However, only a third of Canadian children walk or bike to school, and longitudinal analyses indicate that the likelihood of using active transportation to school increases until age 10 and then decreases [7]. While it may not be possible for all children to walk to school, many Canadian parents who drive their children would allow them to use active transportation if traffic danger were reduced [8]. In particular, high perceived traffic danger on the school route lowers the odds of frequent walking by 47% [9]. As parents control the choices of school travel mode for children, it is essential to consider both actual and perceived collision risk in relation to the built environment. The analysis of these perceptions, both in children and their parents, leads to a better understanding of school travel choices. Active transportation rates are unlikely to improve without action to reduce traffic related injury risk.

Pedestrian and bicycling injuries are among the leading causes of death and hospitalization for Canadian children and youth [10]. Every year in Canada, 30 child pedestrians and 20 child bicyclists are killed and an additional 2400 child pedestrians and 1800 child bicyclists are injured [11, 12]. Based on 2010 data, child and youth pedestrian and bicycling injuries cost Canadians an estimated $266 million annually (T. Walji. Parachute, email communication, Dec. 22nd, 2015). Disability related to pedestrian and bicyclist-MVC is higher than that of other injury mechanisms (e.g., sports related injuries) six months post injury [13]. Research has shown a 4-fold increase in severe bicycling injury risk in children and youth with MV involvement [14]. Child specific studies are needed, as previous work shows that the location of child pedestrian injuries differs from other ages [15].

Many municipalities implement traffic calming strategies and broader infrastructure and built environment approaches, with the hope of shaping active transportation behaviours and road safety [16–18]. Modifying the built environment to promote active transportation can be costly and spark considerable debate at the municipal level. In fact, very little is known about the real efficacy of built environment interventions at promoting active transportation or preventing active transportation injuries [19]. In addition, children (who are still developing physically and cognitively) may have very different traffic infrastructure needs, compared with adults, due to different activity patterns, schedules and destinations. Built environment risk factors for active transportation injuries will also vary by age [15]. Parachute, Canada’s national injury prevention organization, maintains that children under age 10 are not physically and cognitively ready to bicycle on the road with motor vehicle traffic [20] and research supports this recommendation [14]. Child and youth pedestrians face similar challenges. Evaluation of initiatives to increase child and youth active transportation have generally focused on active transportation outcomes, such as participation rates, with little consideration of safety outcomes.

A systematic literature review indicated that bicycle-specific infrastructure (e.g., bike lanes and bike paths) have lower injury and crash risk, while multilane roundabouts without bike lanes, sidewalks, multi-use trails and major roads have higher risk [21]. Despite these important findings, unclear definitions and groupings of infrastructure types make it difficult to interpret injury and crash risk of specific infrastructure features. In addition, the range of infrastructure types studied to date is small compared with the range of existing roadway configurations [21]. Previous bicycling injury research has not adequately measured exposure to risk or controlled for potential confounders [21]. Recent emergency department (ED) based all-ages bicyclist injury research addressed many of these concerns and found significant associations between injury risk and certain infrastructure types (e.g., major streets with parked cars, construction, path obstructions, downhill grades, intersections) [22–24]. These approaches require focused application on child and youth bicyclists given their physical and cognitive differences from adults.

The effectiveness of traffic design interventions has often been examined for adult pedestrians [25, 26], but little is known about children and youth. Recent work found age-related differences in the geography of pedestrian-MVCs specifically related to Toronto’s urban versus inner suburban neighbourhoods, suggesting that urban form and built environment risk factors vary with age [15]. A systematic literature review examining the built environment features associated with safe walking in children found that the majority of traffic design interventions had inconsistent associations with either walking or injury, potentially due to diverse outcomes and built environment measurement [27]. In addition, the majority of studies included in that systematic review were cross-sectional.It is recommended that future research include objective measurements, such as observational counts for walking, and geographic information systems data-based built environment measures. Further, as randomised trials are difficult to implement for traffic interventions, the authors recommended case-control, case-crossover, and quasi-experimental designs with spatial analysis methods. We have studied built environment features associated with child pedestrian-MVCs and walking to school in Toronto and Montreal using environmental audits with cross-sectional [28–31] and pre-post installation quasi-experimental designs [32]. Across these studies, several urban design features (e.g., population density, traffic calming, school crossing guards, one-way streets) have been consistently associated with rates of child pedestrian injuries.

The urban built environment can confer health and health equity benefits (through promoting active transportation) and is an important association of traffic injury, the leading cause of child death. Children differ from adults in both active transportation behaviour and traffic injury risk. The substantial gap in child-specific knowledge can be addressed by this team of researchers and community partners, who build upon their own proven innovative methods. Therefore, the purpose of this research program is to determine how the built environment influences child and youth active transportation and their risk of active transportation injury across different Canadian urban settings.

## Research program objectives

**Fig. 1:**
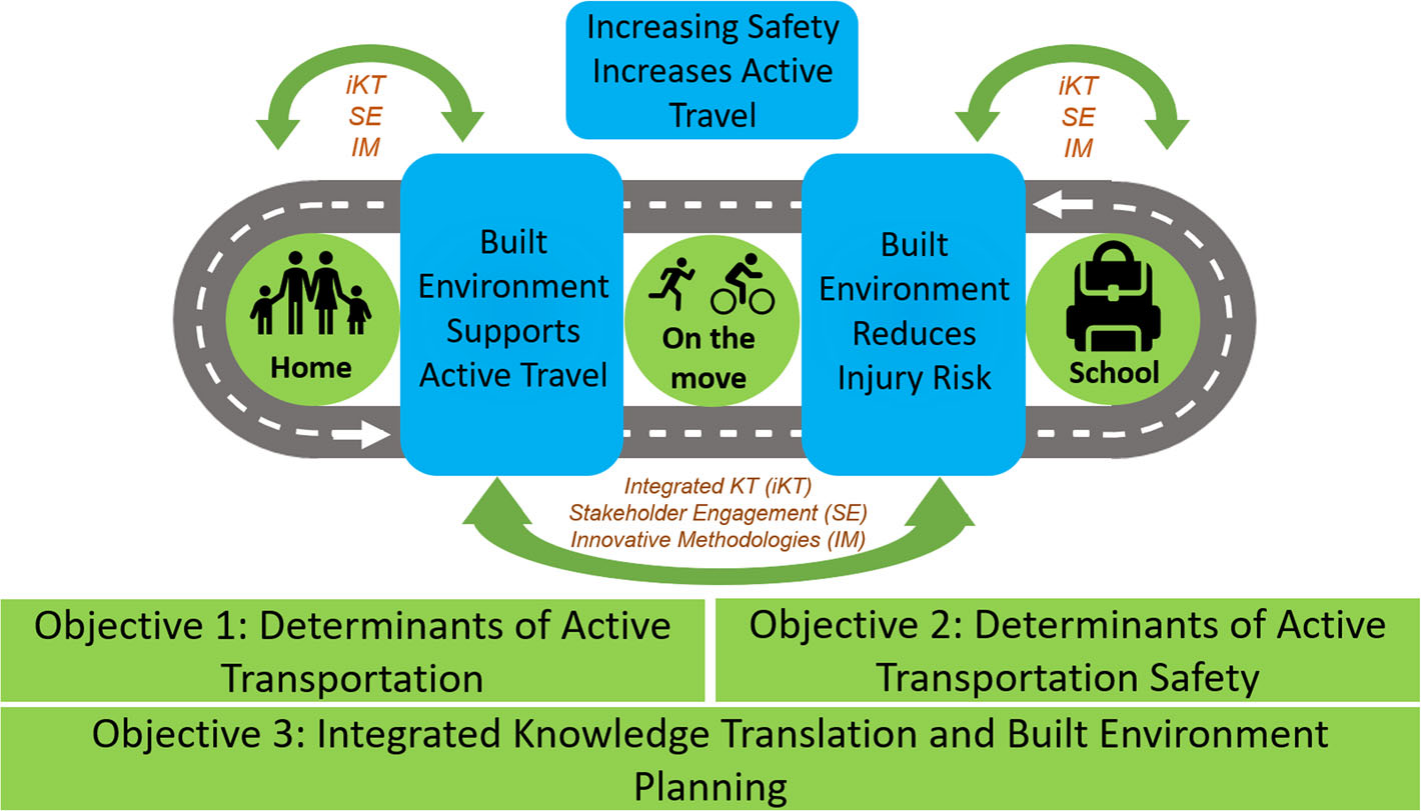
Framework for Built Environment and Active Transportation Safety in Child and Youth Activities

Figure 1illustrates the conceptual linkages between our objectives.

### Objective 1

To examine associations between the built environment and child active transportation to school within and across multiple large Canadian centres

### Objective 2

To examine associations between the built environment and child and youth active transportation injuries in multiple large Canadian centres

- Project 2A: To estimate the associations between the objectively measured built environment and child pedestrian-MVCs and bicyclist-MVCs near schools.
- Project 2B: To analyse the space-time distribution of pedestrian-MVCs and bicyclist-MVCs to understand the geography of child and youth injury events and risk factors.
- Project 2C: To estimate the effect of built environment traffic features on pedestrian-MVCs and bicyclist-MVCs using a quasi-experimental design.
- Project 2D: To estimate the associations between the built environment and child and youth bicyclist-injuries using a case-crossover design.

### Objective 3

To identify implementation strategies for built environment change at the municipal level to encourage active transportation.

- Project 3A: To identify the facilitators and barriers for implementing built environment change at the municipal level.
- Project 3B: To develop a built environment implementation toolkit, adapted for different municipalities, in order to promote active transportation and/or prevent child pedestrian and bicycling injuries.

## Methods/Design

### Objective 1

To examine associations between the built environment and child active transportation to school within and across multiple large Canadian centres

### Study design

A cross-sectional design will be used that will include primary data collection of active transportation around schools and data from secondary sources related to the built environment, social and individual factors.

### Location and participants

A sample of 125 elementary schools (depending on the number of eligible schools in the jurisdiction) will be chosen in four urban regions (Montreal, Toronto, Calgary, Vancouver). Regular program elementary schools (typically Kindergarten-Grade 8) will be included. Schools with older grades will be excluded as older students have very different school transportation patterns, in part due to developmental skills and larger attendance boundaries for middle and high schools. Schools focused on special programs such as arts-based curricula, French immersion, and special needs will be excluded as their students travel further and may be more likely to be driven. Eligible schools will be stratified by Walk Score® (related to population density and urban form) and socioeconomic status (SES; After Tax-Low Income Cutoff [AT-LICO]) in order to represent several urban and suburban built environment types and to enable some exploration of active transportation and SES [31, 33]. Local knowledge users may participate in selecting municipalities and schools. Across each selected SES/built environment stratum, the list of schools will be randomly ordered, and schools will be observed in the order of randomization until the maximum sample in each municipality is met.

### Recruitment

Schools will be recruited for participation as per specific school district policies.

### Data collection

Direct observations of active transportation to school will be conducted, as per published methods [28, 29, 34–36]. Two trained observers will count children’s travel mode (e.g., walking, bicycle, scooter, vehicle) to school, complete a site survey, and detail built environment features on road segments near the school. Vehicle speed and volume will be measured using municipal speed tubes. The site survey will include presence of designated drop-off areas and presence of dangerous driving and pedestrian/bicyclist behaviours using a checklist we have used previously [37]. We found high test-retest reliability of these count observations in Toronto (Pearson’s r = .96) [29]. Some covariates will come from the site survey whereas others will be extracted from a spatial database compiling built environment metrics at the school-level using different network distance buffers (e.g., between 500 m and 1.6 km, the most frequent distances in the literature and representing a credible range of child walking distances). Using different buffer distances addresses the modifiable areal unit problem [38–40]. The problem reflects how scale and zoning affects the measurement of the built environment. For example, using a smaller distance buffer may produce unstable results, whereas a larger distance buffer may increase the correlation of different built environment variables (scale effect). A zoning effect exists when different buffer sizes produces dissimilar results [38]. Conducting a sensitivity analysis using different buffer sizes will help inform the accuracy and stability of our results. Metrics to populate objectively measured built environment predictors (3D’s- density, diversity, design) will be calculated from the spatial databases. Each predictor will be assessed within street network based distance buffers around schools:

#### Density

Population density, housing density.

#### Diversity (urban built environment)

Land use diversity index (entropy), park area (% of the total land), date of housing construction, number of recreational facilities.

#### Design

1) **Road infrastructure**: road length, proportion of each road type per zone (arterial, local streets), one-way streets, sidewalks, separated paths, intersection, roundabouts, traffic circles, dead-ends/cul-de-sacs, transit types, transit stops; 2) **Traffic features**: traffic calming devices (e.g. speed bump, corner radius, chokers, diverters), traffic lights and other signage (stops, yield) density, crosswalks, countdown timers; 3) **Socio-demographic context**: deprivation index and proportion of low-income families, immigrant population, linguistic composition; 4) **School context**: school population, number bussed per school, presence of any active transportation to school program, distance allowance to walk to school (dichotomous), presence of school crossing guards, number of other schools within buffer (i.e. private, catholic), date of school construction, school drop off zone (on or off street), vehicle speed, driving behaviours, pedestrian/bicyclist behaviours.

### Data analysis

The primary outcome variable is the observed proportion of students arriving at school via active transportation. Direct observations of active transportation to school will be used to generate a numerator (students observed walking, bicycling, and other active transportation). The denominator is total observed non-school bus student arrivals (walking, bicycling, arriving by vehicle, and other). Children arriving by school bus will be excluded as they typically live further than walking distance. With the school as the unit of analysis, active transportation proportions will be modeled with built environment predictors using multivariable regression [41]. Sensitivity analysis will use different street network buffer distances. Categorical variables will be included in the model representing each of the sites (municipalities) to enable inter-jurisdictional comparisons. Separate models will also be estimated for each municipality.

### Sample size

The target sample size is 125 schools per municipality. Walking rates by school varied from 28 to 98% with a mean of 67% in our previous studies [29]. Using walking rates as an outcome and using built environment predictors we have measured before, only 50 schools per municipality are required to achieve 95% power at a .05 significance level [42]. We target a larger sample required for an injury model.

### Anticipated concerns

We will keep track of characteristics of non-participating schools. However, our experience to date has been positive with schools keen to participate and the number of non-participant schools has been very low (0/24 schools in Vancouver and 8/126 = 6% in Toronto) [29, 34].

#### Objective 2

To examine associations between the built environment and child and youth active transportation injuries in multiple large Canadian centres

#### Project 2A

To estimate the associations between objectively measured built environment and child pedestrian-MVCs and bicyclist-MVCs near schools

### Study design/location/participants/recruitment

This project has the same study design, locations, and participating schools as Objective 1, with the outcome variables being injury-related. Regression models will use the same predictors, with the addition of a variable for observed active transportation to school, estimated from Objective 1.

### Data collection

Collisions involving child pedestrians and bicyclists, ages 4–12, will be extracted from the most recent ten years available of police reported child pedestrian-motorvehicle collisions (MVCs) and bicyclist-MVCs in each municipality. Our partners in each municipality will facilitate the access to these datasets. The file will include the geographical coordinates of the collision, which will be mapped onto school buffers. Collision rates will be estimated using census populations within school buffers. Using 10 years of collision data together generates stable, interpretable child pedestrian collision rates as demonstrated in our published work from Toronto [28].

### Data analysis

As in Objective 1, the association between child pedestrian-MVCs and bicycle-MVCs and potential built environment predictors will be assessed using multivariable Poisson regression analysis. Alternative modelling (e.g. negative binomial, zero inflated Poisson) will be applied if over-dispersion of the response variable exists. The proportion of children using active transportation to school will be included as a predictor variable to account for exposure. Municipality will be included in the model as a covariate and confounding and effect modification of the other predictors, by municipality, will be evaluated using standard techniques.

### Sample size

Our Toronto work showed an average baseline collision rate of 7.4/10,000 children per year in a sample of 118 elementary schools [28]. Based on this work, traffic calming density had an important association with collision rates with a rate ratio of 1.31 (1.06–1.63) and can be considered an example covariate of interest on which to base our sample size calculations. Traffic calming density had a Poisson distribution and a lambda of 0.44. Therefore, a sample of between 120 and 140 would be required to achieve 95% power at a 0.05 significance level [43]. The existing standard is that there be at least 10 cases for each parameter estimated in a regression model. Our previous model from Toronto had 7 covariates, necessitating at least 70 participant schools. Therefore, by collecting data at up to 125 schools in each of the municipalities, we have sufficient power to detect important rate ratios and the opportunity to compare built environment covariates across geographic locations [42].

### Anticipated concerns

It is possible that policies used by police attending incidents vary between cities. To address this concern, we will do a sub-analysis of collisions classified as serious or fatal. Research suggests more accurate reporting of serious and fatal injury by police [44–46]. It is also possible that we will not have the collision data from the same time period for each municipality. We will deal with this by basing models on the most recent years when data are available from all municipalities.

#### Project 2B

To analyse the space-time distribution of pedestrian-MVCs and bicyclist-MVCs to understand the complex geography of child and youth injury events and risk factors

### Study design/location/participants/recruitment

Spatial density and space-time clustering analyses will take place to assess the dispersion, concentration and localisation in space and time of all child and youth pedestrian-MVCs or bicyclist-MVCs in the four municipalities in Project 2A.

### Data collection

As in Project 2A, we will use the most recent 10-year period of available police-reported traffic collision data. This project will be conducted municipality-wide, not focussed specifically on school zones as in Project 2A.

### Data analysis

Spatial density analyses (area and road network) will be based on three different outcomes: 1) Counts of collisions per census dissemination area by municipality; 2) Counts of collisions per intersection; 3) Counts of collisions per road segment length. Kernel density (area) and network kernel density (road segment) analysis will be used [47–50]. Local Indicators of Spatial Association and Moran’s I will be used to identify hot spots by dissemination area at the municipal and national level [51]. These results will pinpoint potential hot spots of pedestrian-MVCs or bicyclist-MVCs within and between municipalities across Canada and will examine built environment variables within those hot spots. Space-time clustering analysis will be based on two outcomes: 1) Counts of collisions per dissemination area per month by municipality with child population within the dissemination areas considered as the population at risk; 2) Counts of collisions per school zone, with the population at risk being active transportation counts (from Objective 1). Results will be mapped and analysed statistically.

### Anticipated concerns

There is the potential that too few crashes will lead to non-significant results in the spatial cluster analysis since the method is pointing at spatial over-representation. This is, however, less likely to be an issue as we are using ten years of historical collision data from 4 cities and we had sufficient power to detect modest associations in our previous work (Toronto) [28].

#### Project 2C

To estimate the effect of built environment traffic features on pedestrian-MVCs and bicyclist-MVCs using a quasi-experimental design

### Study design

A quasi-experimental design will be used that will include the analysis of collision rates, pre and post traffic feature implementation.

### Location and participants

We will identify the location of newly installed (i.e., within the past ten years) traffic features in four municipalities (Montreal, Toronto, Calgary, Vancouver). Only traffic features with known implementation dates will be included in the study.

### Data collection

We will use the most recent 10-year period of police-reported traffic collision data. This project will be conducted municipality-wide, not focussed specifically on school zones as in Project 2A. Police reports of pedestrian-MVCs and bicycle-MVCs include the geographical coordinates of the collision, which will be mapped for the four municipalities. Collisions will be assigned to a feature either within a 30 m buffer-zone for point features (e.g. traffic lights) [32, 52–54] or within a 25 m buffer zone if along a roadway segment (e.g. traffic calming) [55–57]. As in other projects, potential built environment predictors will be extracted from the spatial database as described in Objective 1. The potential traffic features include (but are not limited to): 1) Traffic calming (including speed humps, chicanes, pinch points, etc.); 2) Changes in speed limits; 3) Curb extensions; 4) Traffic lights; 5) Stop signs; 6) One-way streets; 7) Level 2, uncontrolled pedestrian crossovers in residential areas; 8) Crossing guards; 9) Dead end roads; 10) Cycle lanes and cycle tracks. Location and installation dates for these built environment features will be obtained from municipality transportation departments where possible.

### Data analysis

The analysis will start with descriptive statistics of each feature and pedestrian injury rates in each municipality (Vancouver, Calgary, Toronto, Montreal). We will model collision frequency pre/post installation of each built environment feature, in each municipality, stratified on active transportation mode (pedestrian/bicyclist). The outcome will be the collision rate per feature/month (time as the offset variable). A repeated measures Poisson regression (where the feature’s site acts as its own control) will be used to model rates of pedestrian-MVCs and bicyclist-MVCs pre and post installation of the feature, controlling for feature covariates. The pre-installation period without the feature will be designated as the reference value. For features that are implemented/installed along a length of roadway (e.g., speed limits, traffic calming) we will measure collision rates using incidence density/roadway meter/month. This rate will reflect the number of collisions per meter of roadway that had the feature. For those variables that are implemented/installed at a specific point (e.g. crossing guards, crosswalks) collision rates will be measured using incidence density/traffic feature/month.

### Spatial analysis

This description and inference involves spatial point pattern analyses. There are two levels of analysis to consider: 1) The broad pattern of events across each study area–a consideration of first order effects (variation in the mean of a spatial process); and 2) The extent to which the events cluster or interact with one another, and around specific objects located in the built environment (second order effects). In this study, analysis of second order effects will focus on the clustering of events around each site pre and post installation. For example, a speed hump may displace vehicle flow to neighbouring streets and conversely a bicycle lane may draw bicycle flow from neighbouring streets. In either case, an area analysis is required to fully understand safety consequences both at and around the feature.

### Inter-municipality comparisons

After examining the effects of each feature within each municipality, we will conduct a similar analysis across all cities, using a categorical variable representing each municipality as one of the covariates. This will allow us to estimate the effect of the feature in different settings, and to assess which features are most effective in which context.

### Anticipated concerns

It can be difficult to obtain implementation/installation dates for some traffic features [32]. We will focus on traffic features that we are able to obtain sufficient numbers with installation dates. Details of sample size and time pre and post installation will differ for each traffic feature and for each municipality; however, the feasibility of this analysis is supported by our published results from using this approach to estimate the effectiveness of pedestrian countdown signals [52], speed humps [55], school crossing guards [32], cycle lanes [56], and a streetcar right-of-way, in Toronto [57].

#### Project 2D

To estimate the associations between the built environment and child and youth bicyclist-injuries using a case-crossover design

### Study design

We will use a case-crossover design to compare the built environment characteristics of the site where the injury event occurred (injury site) to those at two randomly selected sites (control sites) along the bicyclist’s route before the injury occurred. Our approach uses the design of a study of infrastructure and bicycling injury risk in adults [24]. It overcomes methodological limitations of previous bicycling injury research by controlling for important confounding by personal, weather, and daylight characteristics by using injured bicyclists on a single trip as their own controls, and addressing exposure to injury risk by comparing injury sites to control sites on the route bicycled on the injury trip.

### Location and participants

The study will be conducted in three municipalities, providing a wide range of infrastructure, geography, and population. Participants will be children and youth (5–17 years old) injured while bicycling and seen at the Emergency Departments (EDs) of the main children’s hospitals in the cities (BC Children’s Hospital-Vancouver; Alberta Children’s Hospital-Calgary; Hospital for Sick Children-Toronto).

### Recruitment

We have an ED physician research team collaborator at each site. These site-investigators are members of Pediatric Emergency Research Canada; a network of clinicians performing multi-centre research projects. A research assistant will be at each site to work with the physician research team to plan and execute study recruitment that aligns with the local situation. A member of the healthcare team or ED research team will provide eligible participants with a study consent to contact/consent form. Study personnel will collect these forms regularly. If time permits and written consent is given by the bicyclist/parent, data collection may occur in the ED. Based on our previous ED research [14], we anticipate that most interviews will occur after the bicyclist is discharged, in which case an in-person interview will be arranged at the bicyclist’s home, hospital, or other convenient location. A written study assent form (for children) and/or consent form (for parents or older children/youth) will be completed before the interview. If an in-person interview cannot be arranged, a telephone interview along with verbal consent may be conducted. We will make a maximum of six call and five email attempts to contact injured bicyclists/parents at different times and on different days consistent with our previous research [14]. Obtaining some information by phone will reduce the likelihood of selection bias resulting from excluding bicyclists not interviewed in person. We have collected and mapped routes over the phone in previous work [58].

Given the focus on bicycling for transportation and the built environment, we will exclude (based on the Teschke et al. [24] study) those: 1) Who live or were injured outside of the participating cities; 2) With no known address or phone number; 3) Fatally injured; 4) Unable to communicate due to injury and lack of parental knowledge about route/crash details; 5) Who do not, and parents do not, speak English; 6) Injured on private property, 7) Injured while trick riding, mountain biking, racing, or riding in a mass bicycling event; 8) Who were riding a motorized bike, unicycle, tricycle, or tandem bike; and 9) Who had previously participated in the study. We will also exclude those who were not contacted and recruited within 3 months of the injury to decrease the likelihood of recall bias. We will keep a detailed account of the reasons for exclusion. We will not exclude those unable to remember all injury trip details, but will obtain as much information as we can about their personal and trip characteristics to assess the magnitude of any potential bias and to use in a sensitivity analysis whereby missing data are imputed.

### Data collection

#### Interview and site selection

We will train a research assistant to conduct the interview using a semi-structured questionnaire to map the route of the injured bicyclist on the injury trip, including any trip continuation after the injury event. Detailed paper maps (including streets and off-street paths) will be used to identify the trip route and injury site. Using a digital map wheel, we will estimate the trip distance and multiply it by two randomly generated proportions to select two control sites along the route. The second of these points will be moved either forward or backward along the route (depending on participants study specific identification number) to match the intersection/non-intersection status of the injury site. We will ask questions on the circumstances of the injury event and characteristics of the injury and control sites on the trip such as street surface conditions, light conditions, presence of construction, and estimated speed. We will also collect data on personal characteristics (age, sex, bicycling experience etc.) and trip characteristics (type of bicycle, clothing colour, weather, time of day) for secondary analyses; the case-crossoverapproach inherently controls for these factors.

We will use the interview procedures and data collection forms that Teschke et al. used with adult bicyclists [24]. Our collaborators with expertise in child and youth injury prevention will modify the interview questions to ensure they were suitable for children and youth. The questionnaire and modifications were pilot tested with additional revisions made prior to initiation of the project.

Given that some children and youth will have mild to severe brain injuries, and that some information may come from very young children, the interviewer will record the source of the interview (child, parent, other proxy respondent, etc.) and rate their confidence in the quality of the interviewee’s description and recall of the bicycling route and site characteristics. We will conduct sensitivity analyses based on the interviewer’s trust in the information quality.

#### Site observations

A trained research assistant blinded to injury/control site status will visit the injury and control sites to conduct structured site observations using a site observation form. Additional research assistants will be available in each municipality in the summer (May–August: high volume for paediatric ED bicycle injuries). Observations will be made as close as possible to the time of the injury event (i.e., season, time of day, weekday versus weekend). The information captured will vary according to route type (roadway, off-road, intersection, etc.). For example, off-road site details include type of path (e.g., pedestrian only, multi-use), surface characteristics (e.g., asphalt, grass), one- or two-way traffic, posted bicycle speed limits, etc.; on-road site details include one or two-way motor vehicle traffic, parking, etc. Intersection details include traffic control devices, intersection features, etc. In addition, we will capture general site details such as slope (Clinometer with Percent and Degree Scales), route visibility (measuring wheel), and the average speed of up to 5 motor vehicles (velocity speed gun). The site observation form will restrict most observations to directly at the site (within 5 m), but some items refer to nearby areas (e.g., features present on the road (within 50 m). The site observation approach has undergone extensive reliability testing with site feature Kappa values of 0.73 to 1.0 [24].

### Data analysis

Route types will be classified into categories consistent with Teschke et al. [24] We will compare personal characteristics by age group: child (< 13 years old) and youth (13–17 years old) consistent with age categories used in our prior bicycling injury research [14, 59]. We will compare the characteristics of the injury and control sites accounting for the fact that we have matched data (i.e., the same subject contributes the injury and control sites along a given route) [60]. We will use conditional logistic regression analysis to examine the route characteristics related to bicycling injuries. We will add no more independent variables than 10% of the number of discordant pairs (case site location characteristics different than control site location characteristics) [61]. We have used this analytic approach in our previous work [62]. Multiple imputation analysis will be used to assess the influence of missing data on effect estimates [63].

#### Sample size

Based on our prior work [14, 64, 65] approximately 49% of bicyclists will meet eligibility criteria (injured in an urban location) and will agree to participate. Public Health Agency of Canada injury surveillance data [66] for 2013 indicated there were 1139 child and youth bicycling injuries at the study EDs (Email Communication, Steve McFaull, Injury Section, Public Health Agency of Canada, June 29, 2016). This number would result in approximately 837 (1139*49% = 558 *1.5 years) eligible injuries from May, 2017-October, 2018. With a sample of 837 injury and matched control sites for each individual, we will be able to detect odds ratios as low as 1.5 with 80% power (0.05 significance level) for a control site exposure prevalence ranging from 10% through 70% [67]. Of note, in the study by Teschke et al. [24], the prevalence of various infrastructure types ranged from 1% (major street with parked cars, shared lane) to 33.5% (downhill grade).

#### Anticipated concerns

Child/youth ability to remember route information may vary. We will have a rating of the interviewer’s confidence in the child/parent description of the site and will also compare this information with site observation data. We will use multiple imputation methods for missing data.

It is possible that some site characteristics may change after the injury (e.g., construction sites). We would expect this potential misclassification to affect both injury and control sites equally, resulting in a bias to the null. We will also ask our municipal partners whether changes were made to these sites.

##### Objective 3

To identify implementation strategies for built environment change at the municipal level to encourage active transportation

##### Project 3A

To identify the facilitators and barriers for implementing built environment change at the municipal level

### Study design

We will conduct a multi-case, mixed-methods study in order to identify facilitators and barriers for implementing built environment changes affecting child pedestrian and bicyclist safety and active transportation at the municipal level. To do this, we will conduct: 1) An environmental scan (that includes a literature review), 2) Key informant interviews, 3) Focus groups, and 4) A national survey. We will conduct an environmental scan of publicly available information (e.g. information gathered from a literature review and municipal documents that outline processes for built environment) and use the synthesized research information from literature reviews of effective built environment interventions. This will inform the development of interview guides for key informants and focus groups and for the national survey.

### Location and participants

We will collect information in environmental scans from each municipality included in this study (Vancouver, Calgary, Toronto, Montreal). Stakeholders involved in the interviews will be representatives from the four municipalities. Key informants and focus group participants will include school staff, school board representatives, school trustees, municipal staff, city councilors, transit authorities, police, provincial transportation authorities, parents, children from each municipality that were/are involved in the implementation of built environment change. Focus group participants include 8–15 stakeholders from each municipality. Focus groups will be used to discuss perceived effects of built environment changes on active transportation and child pedestrian and bicyclist injury prevention, as well as the processes used for case specific built environment changes. Survey participants will include relevant stakeholders from our study municipalities and from other municipalities that have been involved in the implementation of built environment changes. This information will provide the research team with perspectives of broad implementation issues in other municipalities across Canada.

### Recruitment

We will use purposive sampling to recruit 2–3 key informants and 8–15 focus group participants from each municipality (Vancouver, Calgary, Toronto, Montreal), leaving space for increased recruitment until we reach thematic saturation with the data collection. For the on-line national survey, we will use purposive and snowball sampling to recruit participants from municipalities across Canada that have been involved in implementing a school-based built environment change. Written consent will be obtained from all participants.

### Data collection

#### Key informant interviews and focus groups

After consent, data will be collected via 45–60 min semi-structured one on one interviews and focus groups. Interview and focus group guides will follow the Consolidated Framework for Implementation Research [68] and will be used in the development of the questions/discussions, data coding and analysis. The questions will elicit information on: 1) Participant profiles (role, experience, specific built environment intervention implemented, etc.); 2) The implementation process, including the following constructs: intervention characteristics, the outer and inner setting of the organization, characteristics of the individuals involved, the process of implementation, facilitators and barriers; 3) The nature and quality of the approach (practice, policy); 4) Other institutional impacts; and 5) Recommendations arising. Interviews and focus groups will be digitally recorded and transcribed verbatim.

#### Survey

We will develop a national descriptive survey using information collected through our environmental scan, interviews, and focus groups that will be disseminated broadly to municipalities across Canada. The survey will collect data on implementation processes and on facilitators and barriers to the process of implementing a built environment change. The research team will assess content validity and pre-test the survey in a small set of participants. Appropriate changes will be made to the survey before national dissemination.

### Data analysis

#### Key informant interviews and focus groups

Data will be collected and analyzed concurrently, allowing emergent concepts and categories to be incorporated and explored in subsequent interviews/focus groups. We will use qualitative comparative analysis to explore and code clusters of constructs that contribute to the success or failure of the built environment change [69, 70]. We will develop and update a codebook throughout the data analysis process. The codebook will contain code definitions, sample data illustrating application, and decision rules related to each code. Two research assistants will independently code each transcript until consistency is achieved. One coder will use the finalized codebook to code all transcripts. Iterative reduction and clustering of categories will be based on content similarity. Analysis will be performed manually, using qualitative software (NVivo) for data management. The team will review and discuss the findings as they emerge to ensure consistency and authenticity.

#### Survey

We will use descriptive statistics to document the number and type of respondents of the survey, by municipality and type of built environment change, as well as the number and type of indictors/measures/frameworks employed by municipality. We will use content analysis [71] to examine the survey responses and documentary sources. We will use descriptive analysis to categorize the indictors/measures/frameworks used (if any, frequencies with 95% confidence intervals).

##### Project 3B

To develop a built environment implementation toolkit, adapted for different municipalities in order to promote active transportation and/or prevent child pedestrian and bicycling injuries

### Study design

We will use data from the other program objectives, and synthesized information collected in project 3A to develop an on-line built environment implementation toolkit to promote active transportation and/or prevent child pedestrian and bicycling injuries. We will engage participants in a modified Delphi process to develop consensus on the most appropriate format, content, resources and tools for an accessible, on-line toolkit. Information from objectives 1 and 2 will also be compiled into municipality and school/neighborhood level local data reports to be used in conjunction with the toolkit. Knowledge users will be able to request custom reports or visualizations from our spatial databases, and eventually to interact on their own with the proposed web platform and interactive maps. Combining local data with practical and evidence based knowledge translation has been touted as the ‘key to success’ in community-based childhood injury prevention [72, 73].

### Location, participants

Key informants and focus group participants from Project 3A will be recruited for the modified Delphi process. Written consent will be obtained from all participants.

### Data collection

The data collected in Objectives 1, 2, and Project 3A will be used in Project 3B. We will synthesize information collected from environmental scans (including a literature review), key informants, focus groups, and surveys on the facilitators and barriers to implementing built environment changes. An evidence synthesis framework and its associated resources and tools, categorized by built environment change, will be adapted for this study [74]. The framework tabulates contextual information on the built environment change itself (e.g., risk and protective factors, implementation, evaluation). We will also gather information from documents about a built environment implementation strategy or evaluation that was used. The table from the framework will be used as background information for the modified Delphi process.

### Data analysis

We will develop an on-line toolkit to be housed on the previously mentioned research program web platform to assist municipalities on how to modify the built environment to promote active transportation and pedestrian and bicyclist safety. The toolkit will be developed in consultation with all knowledge users and stakeholders and led by Parachute, our national injury prevention organization with both the mandate and experience to create and disseminate community action kits (http://www.parachutecanada.org/injury-topics/topic/C14). A three round Delphi process will be used to gain consensus from knowledge users and stakeholders for the appropriate content and format for the toolkit: Round 1) Information will be provided to participants from data collected and synthesized from the first phase, including local quantitative data from our databases. Participants will respond with suggestions for interventions to be considered; Round 2) Participants will rank order content and tools included in the toolkit (e.g., information on built environment change, links, resources, tools) with reasons for the ranking (to provide data for consensus making); Round 3) We will report and discuss all data, ranking, reasons, minority opinions, and consensus items to finalize information to be included in the toolkit. The research team will review, discuss, and interpret findings from the modified Delphi process in order to finalize the toolkit. The toolkit will also include an evaluation framework.

### Anticipated concerns

Factors associated with implementation may differ among municipalities, and may be related to the built environment change itself, or to the context, including factors such as budget, politics, existing infrastructure, the decision making process, or others. We aim to elucidate information about these factors and plan to include proposed strategies to deal with them in the toolkit. Other anticipated concerns include challenges in finding focus group or interview participants, conflicting information from focus groups or interviews, and challenges such as regulatory changes. We will address these concerns by using our knowledge user community as expert advisors. For example, if one municipality identifies a factor as a facilitator for a built environment change, and another identifies that same factor as a barrier, the toolkit will provide users with information about how each municipality addressed that issue.

## Discussion

Canadian children and youth deserve the health and social benefits of active transportation in a safe environment. Canadian municipalities were built before the strong influences of built environment on active transportation and road injury were understood, leaving considerable variability within and between municipalities in the appropriateness of the built environment. Understanding what works best requires observational and spatial epidemiology. Optimizing the built environment to promote active, safe transportation for children simultaneously confers the health benefit of lifelong protection against most forms of chronic disease, while also reducing risks of injury, the leading cause of death and disability in childhood. Our team’s national scope will be invaluable in providing variability for the study of built environment characteristics and is vital to producing evidence-based recommendations that will increase safe active transportation.

## Data Availability

Not applicable.

## Abbreviations

AT-LICO: After-tax low-incomecut-off
ED: Emergency department
MVC: Motor vehicle collision
SES: Social economic status
